# Oxygenation index outperforms the P/F ratio for mortality prediction

**DOI:** 10.1186/cc13456

**Published:** 2014-03-17

**Authors:** K Davies, C Bourdeaux, T Peiris, T Gould

**Affiliations:** 1 Bristol Royal Infirmary, Bristol, UK; 2 University of Southampton, UK

## Introduction

The P/F ratio is widely used clinically and as part of research to categorise severity of respiratory failure [1]. However, no account is made of an important determinant of oxygenation; mean airway pressure (MAP). The oxygenation index (OI) incorporates the MAP and has been suggested as a more accurate means of determining severity of respiratory failure [2]. In addition, the optimal time for this assessment is unclear. We sought to answer these questions by analysing a large database of patient and ventilator data.

## Methods

The ICU of the Bristol Royal Infirmary has used an electronic clinical information system (CIS) since 2008, with every hour of care available for analysis as a result. Ventilated patients were identified, the P/F ratio and OI were calculated and the worst values for these determined for four time periods (first 12, 24, 36 and 48 hours of ventilation). Logistic regression analysis was used to create models to predict unit and hospital mortality.

## Results

Data for over 150,000 hours of care in 4,886 patients was available for analysis. Excluding nonventilated patients and those transferred ventilated from another ICU, 2,156 patients provided data. In comparison with survivors, nonsurvivors were older, with higher OI and 24-hour SOFA scores and lower P/F ratios. The optimal time for calculation of both OI and P/F ratio for mortality prediction is the first 12 hours of ventilation. The models using worst Ol are better predictors of ICU and hospital mortality than those using worst P/F ratio (area under receiver operating curve 0.840 vs. 0.822).

## Conclusion

Our analysis suggests that the OI is a more sensitive descriptor of the severity of respiratory failure than the P/F ratio and that this calculation should be performed using data from the first 12 hours of ventilation.
